# Association of osteoporosis with sarcopenia and its components among community-dwelling older Chinese adults with different obesity levels: A cross-sectional study

**DOI:** 10.1097/MD.0000000000038396

**Published:** 2024-06-14

**Authors:** Xing Yu, Yaqing Zheng, Yuewen Liu, Peipei Han, Xiaoyu Chen, Naiwen Zhang, Yejia Ni, Ziyi Zhou, Qi Guo

**Affiliations:** a Department of Rehabilitation Medicine, Shanghai University of Medicine and Health Sciences Affiliated Zhoupu Hospital, Shanghai, China; b Department of Rehabilitation Medicine, Shanghai University of Medicine and Health Sciences, Shanghai, China; c Department of Fujian Provincial Hospital, Fujian Provincial Clinical Medical College of Fujian Medical University, Medical, Fujian, China; d Department of Rehabilitation Medicine, School of Health, Fujian Medical University, Fuzhou, China.

**Keywords:** BMI, osteoporosis, sarcopenia

## Abstract

We aimed to investigate whether sarcopenia and its components are associated with osteoporosis in community-dwelling older Chinese adults with different obesity levels. This cross-sectional study included 1938 participants (42.1% male) with a mean age of 72.1 ± 5.9 years. The categorization of individuals into various weight categories was based on the Working Group on Obesity in China’s criteria, utilizing the body mass index (BMI) as follows: underweight, BMI < 18.5 kg/m^2^; normal weight, 18.5 ≤ BMI < 24 kg/m^2^; overweight, 24 ≤ BMI < 28 kg/m^2^; and obesity, BMI ≥ 28 kg/m^2^. In this research, the osteoporosis definition put forth by the World Health Organization (bone mineral density T-score less than or equal to −2.5 as assessed by Dual-energy X-ray absorptiometry (DXA)). Sarcopenia was defined according to the diagnostic criteria of the Asian Working Group for Sarcopenia. The prevalence of osteoporosis was highest in the underweight group and gradually decreased with increasing BMI (Underweight: 55.81% vs Normal weight: 45.33% vs Overweight: 33.69% vs Obesity: 22.39). Sarcopenia was associated with elevated odds of osteoporosis in normal-weight subjects independent of potential covariates (OR = 1.70, 95% CI = 1.22–2.35, *P* = .002). In normal-weight participants, a higher appendicular skeletal muscle mass index (ASMI) was associated with a reduced risk of osteoporosis (OR = 0.56, 95% CI = 0.42–0.74, *P* < .001). In this study, we found that the prevalence of osteoporosis was highest in the underweight group and gradually decreased with increasing BMI. Sarcopenia, body fat percentage, and ASMI were associated with elevated odds of osteoporosis in normal-weight subjects independent of potential covariates, and higher percent body fat (PBF) was associated with an increased risk of osteoporosis in overweight people, and no such association was found in other weight groups. Different amounts of adipose tissue and muscle mass may alter bone biology. Further longitudinal follow-up studies are required to more accurately assess the risk of osteoporosis and sarcopenia in different weight populations. This cross-sectional study found that the prevalence of osteoporosis was highest in the underweight group and gradually decreased with increasing BMI. Sarcopenia was associated with elevated odds of osteoporosis in normal-weight subjects independent of potential covariates.

## 1. Introduction

Osteoporosis, defined as decreased bone quantity and quality, is a major public health concern. Worldwide, one in 2 women aged ≥ 50 years and one in 5 men aged ≥ 50 years will have an osteoporotic fracture in their lifetime.^[1]^ Aging-related hormonal changes, along with decreased physical activity, promote bone resorption and inhibit bone formation. In the elderly population, the risk of fractures is exacerbated by muscle wasting and sarcopenia.^[2,3]^

The World Health Organization defines obesity as the accumulation of excessive fat, which is a growing societal issue with potential health implications.^[4]^ Several studies have suggested that higher body weight may attenuate bone loss during menopause, and the mechanical impact of increased body weight on bones may contribute to the positive effects of obesity on bone mineral density (BMD).^[5,6]^ Garnero et al discovered a reduction in biochemical bone markers among obese individuals, notably a more significant drop in bone resorption markers compared to bone formation markers. Obese people exhibit an increase in both fat mass and lean mass, yet it is the latter that positively contributes to bone density, as evidenced by Gkastaris’ findings.^[7,8]^ The influence of mechanical loading on bone is predominantly determined by lean mass, rather than fat mass.^[9,10]^ However, some researchers have observed an augmentation in bone mass at the lumbar spine, radius, and tibia in obese women, but not in men.^[11]^ While estrogens produced by adipose tissue positively influence bone metabolism by enhancing bone formation and diminishing bone resorption, obesity has been linked to a heightened risk of various diseases and chronic inflammation.^[12]^ Adipose tissue accumulation within the bone marrow can lead to an inflammatory state, disrupting stem cell differentiation and compromising regenerative and immune responses.^[13–15]^ The impact of obesity, as determined by the body mass index (BMI), on bones remains a topic of debate.

The occurrence of osteoporosis is influenced by various physiological mechanisms that underlie sarcopenia and obesity, encompassing alterations in body composition, shifts in muscle fiber types, hormonal level reductions, inflammation, psychosocial factors, the development of joint pain, and a sedentary lifestyle.^[16]^ A strong correlation between osteoporosis and sarcopenia has been suggested by numerous pieces of evidence, stemming from observational studies..^[17,18]^ Some studies suggest a bidirectional association between osteoporosis and sarcopenia.^[18,19]^ However, there are still different conclusions regarding components related to sarcopenia, such as muscle strength and physical function, especially in populations with different obesity levels.^[4,20,21]^ For a long time, it was believed that the age-related decrease in weight, along with the loss of muscle mass, was primarily accountable for muscle weakness and sarcopenia among older people.^[22]^ However, with aging, physical activity decreases, which may lead to weight gain, primarily with an increase in visceral abdominal fat, which leads to sarcopenic obesity.^[23]^ Thus, in this study, we aimed to investigate whether sarcopenia and its components were associated with osteoporosis among community-dwelling older Chinese adults with different obesity levels.

## 2. Methods

### 2.1. Study participants

This cross-sectional study enrolled patients participating in the Adult Physical Fitness and Health Cohort Study (APFHCS) [ChiCTR1900024880]. The APFHCS, a significant prospective dynamic cohort research, primarily delved into the correlation between physical fitness and health conditions in China’s general adult populace. Our study encompassed 2169 senior citizens (aged 60 and above) hailing from Shanghai, China. Each participant underwent an annual health examination and a thorough questionnaire focusing on their lifestyle habits and medical histories. This endeavor adhered to the recommendations of both national and international guidelines, as well as the standards set by our ethical committee.

All participants provided written informed consent by the Declaration of Helsinki. The study protocol was approved by the Ethical Committee of the Shanghai University of Medicine and Health Sciences. The inclusion criteria for participants were age ≥ 60 years and completion of the relevant tests. Exclusion criteria were as follows: those unable to provide signed informed consent; those suffering from severe cognitive impairment, dementia, psychiatric disorders, or other neurodegenerative diseases; those with severe visual and hearing impairments that hindered communication; those unable to stand for the measurement of body composition, weight, and height due to injury, rendering bone density testing unfeasible; and patients who were taking medications that could potentially interfere with bone or calcium metabolism, such as estrogen, calcitonin, and diphosphonate.

A total of 2169 participants participated in the study. Owing to missing values or failure to meet the inclusion and exclusion criteria, 231 participants were excluded. The final analytical sample consisted of 1938 patients (men 816, women 1122). The selection process of participants is shown in the Supplementary Figure, see Figure, Supplemental Digital Content, http://links.lww.com/MD/M791, which illustrates the flow chart of the study.

### 2.2. Assessment of BMD

Portable dual-energy X-ray absorptiometry (EXA-3000; OsteoSys, Co., Ltd., 901-914, 9F, Jnk Digitaltower, 111 Digital-ro 26, Guro-gu, Seoul 152-848, Republic of Korea) was used to measure BMD. The area of BMD (g/cm^2^) was measured at the distal one-third radius of the non-stressed forearm. The daily calibration of the densitometers was carried out by trained technicians using equipment-specific phantoms, and they also conducted all examinations. The World Health Organization’s definition of osteoporosis (BMD T-score less than or equal to −2.5 as assessed by dual-energy X-ray absorptiometry) was adopted for this research.^[24]^

### 2.3. Assessment of obesity

Body composition analysis was performed using direct segmental multi-frequency bioelectrical impedance analysis (BIA) (In-Body720; Biospace Co, Ltd, Seoul, Korea). One hour prior to the assessment, participants were instructed to refrain from eating and consuming excessive amounts of water. BIA furnished definitive measurements for appendicular skeletal muscle mass, fat-free mass, PBF, and total body water. The categorization of individuals into various weight categories was based on the Working Group on Obesity in China’s criteria, utilizing the BMI as follows: underweight, BMI < 18.5 kg/m^2^; normal weight, 18.5 ≤ BMI < 24 kg/m^2^; overweight, 24 ≤ BMI < 28 kg/m^2^; and obesity, BMI ≥ 28 kg/m^2^.^[25]^

### 2.4. Assessment of sarcopenia

The definition of sarcopenia was formulated in accordance with the diagnostic standards established by the Asian Working Group for Sarcopenia (AWGS), encompassing both low muscle mass, as well as low muscle strength and/or poor physical performance, details of measurement methods can be found in our previous study.^[24]^ Muscle mass was measured using direct segmental multifrequency BIA (In-Body720; Biospace Co., Ltd.). Low muscle mass was diagnosed as a skeletal muscle index (ASM/ht^2^) lower than 7.0 and 5.7 kg/m^2^ in men and women, respectively. Muscle strength was evaluated by grip strength using a dynamometer (GRIP-D; Takei Ltd., Niigata, Japan). Participants were asked to exert maximum effort twice using their dominant hand, and the result from the strongest hand was used for the analysis. Low muscle strength was defined as grip strength < 26 and < 18 kg in males and females, respectively. Usual gait speed was used as an objective measurement of physical performance, poor physical performance was assessed by gait speed which was less than 0.8 m/s in both men and women.

### 2.5. Covariates

The standardized questionnaire was administered to all invited participants during a face-to-face interview, aiming to gather data on sociodemographic profiles, health-related habits, and the status of chronic diseases. These factors served as covariates, as outlined in our previous research.^[24]^ Among the sociodemographic factors, we collected information on gender, age, educational attainment, monthly earnings, living arrangements, and marital status. Additionally, the participants’ physical activity levels and sitting durations in the past week were evaluated utilizing the concise version of the International Physical Activity Questionnaire.^[26]^ The assessment of grip strength (in kilograms) was carried out with the aid of a handheld dynamometer (GRIP-D, Takei Ltd). Furthermore, to assess walking speed, 2 sets of laser-based timing devices were positioned at the start and finish of a 4-meter course. The participants were instructed to traverse the 4-meter distance at their regular, steady pace. The medical history was taken to record whether the participants had diabetes mellitus (T2DM), hypertension, hyperlipidemia, cardiovascular disease, pulmonary disease, stroke, etc.

### 2.6. Statistical analysis

The sociodemographic, lifestyle, and health-related characteristics of participants were presented according to the categories of osteoporosis prevalence based on the categorized BMI. When evaluating the disparities in baseline traits, continuous data points were portrayed by their average ± the standard deviation, employing either the *t* test or the Mann–Whitney *U* tests. Meanwhile, categorical variables were expressed as percentages, analyzed through the chi-square test. Logistic regression analysis was used to analyze the relationship between PBF, sarcopenia, appendicular skeletal muscle mass index (ASMI), grip strength, walking speed, and osteoporosis in the study population categorized by BMI. The results are expressed as odds ratios (OR), 95% confidence intervals (CIs), and corresponding *P* value. The confounding factors of sex, age, smoking and drinking habits, living conditions, and history of diseases were adjusted. Statistical significance was set at *P* < .05. SPSS v26.0 (https://www.ibm.com/cn-zh/products/spss-statistics?lang=en_US) was used the statistical analysis.

## 3. Results

**Figure 1.**
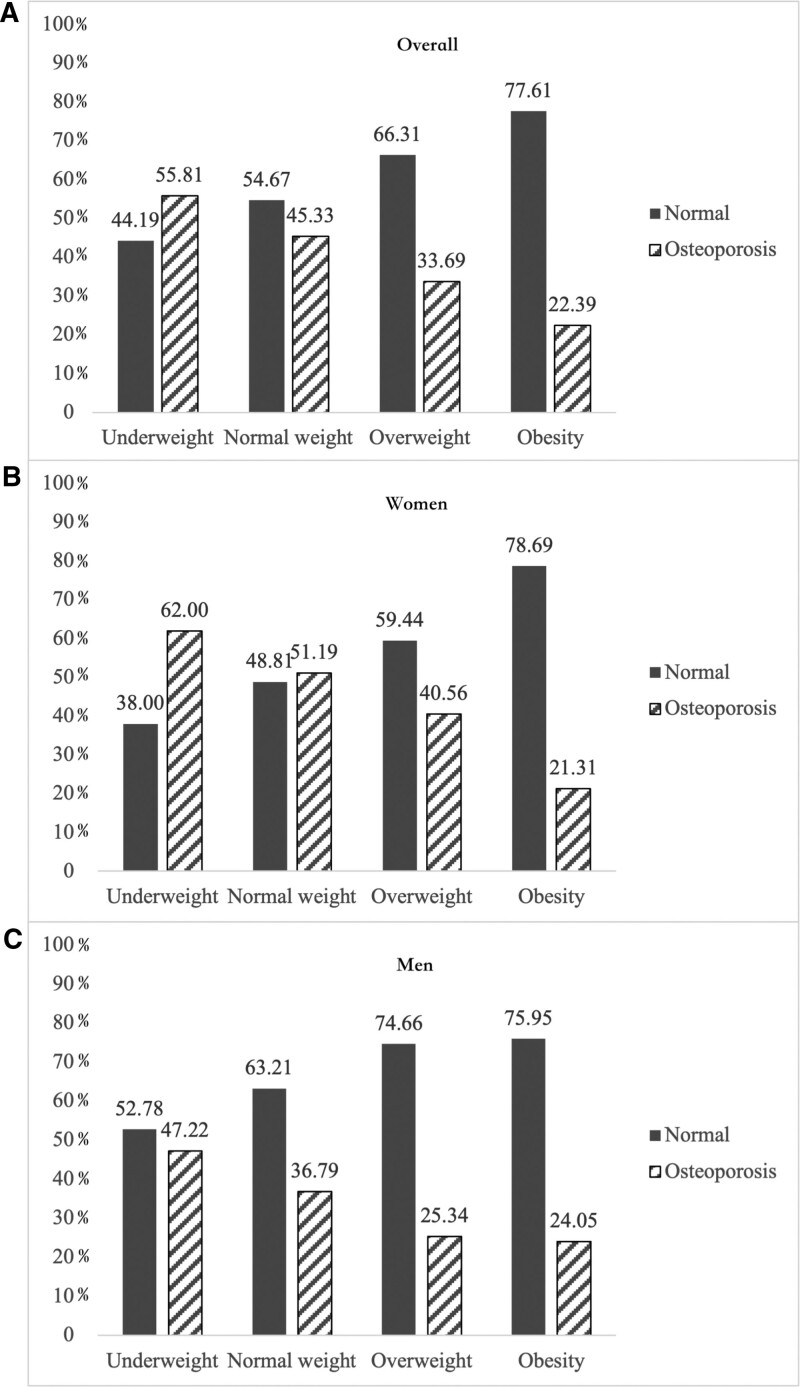
Prevalence (%) of osteoporosis based on the categorized of BMI among the study population.

The final study included 1938 participants (42.1% male) with a mean age of 72.1 ± 5.9 years. The prevalence of osteoporosis was 39.47% in all participants. Overall, 4.4% of the subjects were underweight, 51.3% had normal weight, 33.8% were overweight, and 10.4% were obese. The prevalence of osteoporosis was highest in the underweight group and gradually decreased with increasing BMI, as shown in Figure 1.

The overall characteristics of the study population and according to categories of prevalence of osteoporosis based on BMI are shown in Table 1. In the underweight group, people with lower activity levels and shorter sleep durations were more likely to suffer from osteoporosis (*P* < .05). People with significantly lower muscle mass and higher body fat percentage were more likely to have osteoporosis in the normal-weight and overweight groups and had lower activity levels (*P* < .05). In the obesity group, people with significantly lower walking speed and older age were more likely to develop osteoporosis (*P* < .05). The prevalence of osteoporosis was higher in female subjects in the normal-weight and overweight groups (*P* < .05), as shown in Table 1.

**Table 1:** Characteristics of the study population overall and according to categories of prevalence of osteoporosis based on the categorized of BMI.

Variables	Overall study population	Underweight n = 86		*P* value	Normal weight n = 995		*P* value	Overweight n = 656		*P* value	Obesity n = 201		*P* value
		Normal n* = *38	OP n* = *48		Normal n* = *544	OP n* = *451		Normal n* = *435	OP n* = *221		Normal n* = *156	OP n* = *45	
Age (years)	72.1 ± 5.9	71.45 ± 6.64	73.94 ± 5.55	.062	7.64 ± 5.31	74.02 ± 6.45	<.001	70.74	73.69	<.001	71.21 ± 5.33	76.27 ± 6.65	<.001
Gender				.173			<.001			<.001			.649
Female (%)	57.9	50.0	64.6		52.9	67.0		49.2	66.1		61.5	57.8	
Male (%)	42.1	50.0	35.4		47.1	33.0		50.8	33.9		38.5	42.2	
BMI (kg/m^2^)	23.74 ± 3.34	17.64 ± 0.84	17.36 ± 1.00	.183	21.82 ± 1.49	21.60 ± 1.51	.021	25.74 ± 1.10	25.72 ± 1.06	.824	30.04 ± 2.01	29.56 ± 1.59	.152
ASM (kg)	16.93 ± 4.13	17.62 ± 4.69	15.73 ± 3.26	.227	17.08 ± 4.16	14.58 ± 2.88	<.001	18.10 ± 4.16	16.21 ± 3.43	.003	18.98 ± 4.56	19.38 ± 3.69	.754
ASMI (kg/m^2^)	6.71 ± 1.01	5.85 ± 1.02	5.52 ± 0.87	.101	6.57 ± .89	6.17 ± 0.80	<.001	7.20 ± 1.02	6.82 ± .86	<.001	7.64 ± 1.10	7.60 ± 0.91	.848
BFM (kg)	17.88 ± 6.11	7.46 ± 2.37	7.67 ± 2.82	.718	14.68 ± 3.84	14.93 ± 3.59	.300	20.69 ± 3.41	21.52 ± 3.42	.005	27.85 ± 5.74	26.97 ± 4.01	.370
PBF (%)	28.94 ± 7.45	16.19 ± 6.18	18.01 ± 7.13	.220	25.74 ± 6.34	27.49 ± 5.95	<.001	30.88 ± 5.75	33.68 ± 5.24	<.001	36.72 ± 6.77	36.49 ± 5.65	.847
Grip Strength (kg)	23.55 ± 9.10	23.53 ± 9.50	20.44 ± 6.17	.071	24.73 ± 9.12	21.23 ± 7.54	<.001	26.14 ± 10.02	21.86 ± 8.68	<.001	23.05 ± 9.63	20.78 ± 7.21	.144
WS (m/s)	1.08 ± 0.25	1.11 ± 0.24	1.10 ± 0.23	.743	1.12 ± .21	1.04 ± 0.26	<.001	1.12 ± 0.23	1.01 ± .27	<.001	1.03 ± 0.26	0.90 ± 0.25	.006
WHR	0.91 ± 0.09	0.82 ± 0.06	0.83 ± 0.07	.473	.89 ± .10	0.89 ± 0.11	.513	0.93 ± 0.06	.94 ± .08	.190	0.95 ± 0.71	0.97 ± 0.07	.175
IPAQ (Met-min/wk)	5110 (1386, 6720)	4746 (2053,11466)	3066 (982,5586)	.011	4200 (1533,7812)	3102 (1386,5880)	<.001	4053 (1386,7456)	2772 (1221,4746)	<.001	4053 (1386,7832)	2226 (1386,5434)	.101
Sedentary Time (h/d)	4.23 ± 2.27	4.50 ± 2.67	4.17 ± 2.07	.530	4.08 ± 2.21	4.16 ± 2.28	.593	4.07 ± 2.18	4.71 ± 2.29	.001	4.52 ± 2.56	4.63 ± 2.31	.792
Living alone (%)	13.5	15.8	25.0	.297	11.4	16.9	.013	10.3	15.8	.042	11.5	17.8	.272
Widow (%)	15.5	23.7	12.5	.175	12.7	20.6	.001	11.3	18.6	.010	16.0	20.0	.531
Family Income monthly (%)				.604			.504			.311			.269
<1000	3.9	2.6	2.1		6.1	8.8		5.6	7.7		10.3	8.9	
1000-3000	33.7	47.4	39.6		32.9	32.4		35.2	27.6		31.4	17.8	
3000-5000	15.1	18.4	18.8		14.2	14.6		13.1	14.9		12.8	33.3	
>5000	47.2	31.6	39.6		46.9	44.1		46.2	49.8		45.5	40.0	
Smoking status (%)				.284			.029			.147			.383
Current smokers	12.3	28.9	12.5		16.2	13.5		15.9	14.9		14.7	6.7	
Never smokers	72.7	65.8	79.3		68.6	75.8		66.0	73.3		67.3	71.1	
Past smokers	15.0	5.3	8.3		15.3	10.6		18.2	11.8		17.9	22.2	
Drinking status (%)				.518			.109			.053			.171
Daily drinkers	9.9	15.8	12.5		14.0	9.9		15.9	9.9		14.1	15.6	
Occasional drinkers	16.8	15.8	4.2		16.0	14.6		16.3	19.9		21.8	11.1	
Past drinkers	9.4	7.9	10.4		1.5	8.4		10.3	7.7		5.1	4.4	
Never drinkers	63.9	60.5	72.9		59.6	67.0		57.5	62.4		59.0	68.9	
Sleep Duration(h/d)	7.61 ± 3.02	8.38 ± 3.20	7.21 ± 2.02	.044	7.54 ± 2.92	7.68 ± 3.15	.474	7.55 ± 2.81	7.51 ± 3.41	.881	7.92 ± 3.41	7.55 ± 1.82	.486
Diseases (%)													
Sarcopenia	16.5	44.7	60.4	.148	17.3	31.5	<.001	3.2	8.6	.003	2.6	0	.278
Diabetes	21.1	13.2	12.5	.928	19.7	17.7	.438	19.8	28.1	.016	30.1	35.6	.489
Hypertension	65.6	52.6	47.9	.664	57.4	62.3	.113	71.0	78.3	.047	75.0	82.2	.313
Hyperlipidemia	42.2	28.9	27.1	.509	4.1	36.8	.239	46.0	45.2	.388	54.5	53.3	.622
Gout	6.8	5.3	2.1	.425	5.0	5.5	.682	9.7	5.0	.038	10.9	13.3	.651
Anemia	4.8	10.5	10.4	.987	7.4	4.0	.024	3.4	3.2	.850	1.9	2.2	.899
Depression	12.0	13.2	16.7	.652	11.0	15.3	.046	9.7	11.3	.508	10.3	17.8	.170
Pulmonary disease	9.9	15.8	14.6	.877	1.1	10.2	.963	8.0	14.5	.010	4.5	6.7	.554
Biliary tract disease	15.8	10.5	4.2	.250	12.5	18.2	.013	16.3	19.0	.390	18.6	20.0	.831
Kidney disease	7.9	13.2	14.6	.850	8.1	5.5	.116	8.3	8.6	.888	8.3	8.9	.906
Peptic ulcer	13.4	26.3	14.6	.175	13.6	16.0	.295	12.2	9.5	.305	10.9	13.3	.651
Hepatic disease	12.3	13.2	12.5	.928	9.4	10.4	.581	13.8	12.7	.690	21.8	17.8	.559
Heart disease	27.5	18.4	18.8	.969	26.3	28.8	.372	29.2	25.8	.359	28.8	33.3	.562
Thyroid disease	7.6	7.9	12.5	.488	6.8	7.8	.561	7.6	9.0	.516	7.1	4.4	.531
Osteoarthritis	15.1	10.5	10.4	.987	12.1	16.6	.043	15.6	16.7	.714	16.7	24.4	.236
Cancer	3.4	2.6	6.3	.429	3.9	2.7	.293	2.8	4.1	.366	3.2	6.7	.295

BFM = body fat mass, BMI = body mass index, IPAQ = International Physical Activity Questionnaire, PBF = percent body fat, SMI = skeletal muscle index, SMM = skeletal muscle mass, WHR = waist-to-hip ratio, WS = walking speed.

Results of the logistic regression analyses for osteoporosis with sarcopenia and its components in underweight subjects are shown in Table 2. After adjusting for potential confounders (sex, age, smoking, drinking habits, living conditions, and history of diseases), we did not observe significant differences in underweight subjects with and without osteoporosis.

**Table 2:** Logistic regression analyses for osteoporosis with sarcopenia and its components in underweight subjects.

	Variables	Crude		Model 1		Model 2	
		OR (95% CI)	*P* value	OR (95% CI)	*P* value	OR (95% CI)	*P* value
Underweight Cases n* = 86* OP Cases n = 48	Sarcopenia	1.89 (0.80–4.47)	.149	1.57 (0.64–3.89)	.327	1.16 (0.21–6.33)	.861
	PBF (%)	1.04 (0.98–1.11)	.219	1.02 (0.91–1.13)	.794	1.01 (0.89–1.14)	.878
	ASMI (kg/m^2^)	0.66 (0.39–1.09)	.105	0.90 (0.43–1.92)	.793	1.07 (0.88–1.31)	.481
	Grip Strength (kg)	0.95 (0.89–1.01)	.080	0.97 (0.90–1.04)	.338	0.99 (0.91–1.19)	.892
	WS (m/s)	0.73 (0.11–4.73)	.739	1.50 (0.20–11.36)	.698	2.44 (0.22–27.15)	.467

Model 1 was adjusted for gender and age. Model 2 was adjusted for Model 1 variables, in addition to smoking and drinking habits, living conditions, physical activity, marital status and history of diseases.

BFM = body fat mass, PBF = percent body fat, SMI = skeletal muscle index, SMM = skeletal muscle mass, WS = walking speed.

Results of the logistic regression analyses for osteoporosis with sarcopenia and its components in normal weight subjects are shown in Table 3. After adjusting for potential confounders, we observed that sarcopenia (OR = 1.70, 95% CI = 1.22–2.35), PBF (OR = 1.59, 95% CI = 1.02–1.10), and ASMI (OR = 0.55, 95% CI = 0.42–0.74) were significantly correlated with osteoporosis in normal-weight subjects.

**Table 3:** Logistic regression analyses for osteoporosis with sarcopenia and its components in normal weight subjects.

	Variables	Crude		Model 1		Model 2	
		OR (95% CI)	*P* value	OR (95% CI)	*P* value	OR (95% CI)	*P* value
Norma l Weight Cases n* = 995* OP Cases n = 451	Sarcopenia	2.20 (1.63–2.96)	<.001	1.64 (1.19–2.26)	.003	1.59 (1.13–2.24)	.008
	PBF (%)	1.05 (1.03–1.07)	<.001	1.06 (1.02–1.10)	.001	1.06 (1.02–1.10)	.007
	ASMI (kg/m^2^)	0.56 (0.48–0.66)	<.001	0.57 (0.43–0.75)	<.001	0.90 (0.85–0.96)	.001
	Grip Strength (kg)	0.95 (0.94–0.97)	<.001	0.98 (0.96–1.00)	.061	0.98 (0.95–1.00)	.066
	WS (m/s)	0.22 (0.13–0.38)	<.001	0.61 (0.33–1.13)	.119	0.62 (0.32–1.22)	.168

Model 1 was adjusted for gender and age. Model 2 was adjusted for Model 1 variables, in addition to smoking and drinking habits, living conditions, physical activity, marital status and history of diseases.

BFM = body fat mass, PBF = percent body fat, SMI = skeletal muscle index, SMM = skeletal muscle mass, WS = walking speed.

The results of the logistic regression analyses for osteoporosis with sarcopenia and its components in overweight and obese subjects are shown in Tables 4 and 5. After adjusting for potential confounders, we observed that PBF (OR = 1.09, 95% CI = 1.04–1.14) was significantly correlated with osteoporosis in overweight subjects.

**Table 4:** Logistic regression analyses for osteoporosis with sarcopenia and its components among overweight subjects.

	Variables	Crude		Model 1		Model 2	
		OR (95% CI)	*P* value	OR (95% CI)	*P* value	OR (95% CI)	*P* value
Overweight Cases n* = 656* OP Cases n = 221	Sarcopenia	2.83 (1.39–5.76)	.004	1.35 (0.61–2.97)	.454	1.32 (0.59–2.95)	.498
	PBF (%)	1.10 (1.06–1.13)	<.001	1.09 (1.04–1.14)	.001	1.09 (1.04–1.14)	.001
	ASMI (kg/m^2^)	0.66 (0.55–0.79)	<.001	0.88 (0.65–1.18)	.388	1.04 (0.88–1.22)	.682
	Grip Strength (kg)	0.95 (0.93–0.97)	<.001	0.98 (0.95–1.00)	.068	0.98 (0.95–1.00)	.101
	WS (m/s)	0.18 (0.09–0.36)	<.001	0.51 (0.24–1.10)	.087	0.56 (0.26–1.24)	.155

Model 1 was adjusted for gender and age. Model 2 was adjusted for Model 1 variables, in addition to smoking and drinking habits, living conditions, physical activity, marital status and history of diseases.

BFM = body fat mass, PBF = percent body fat, SMI = skeletal muscle index, SMM = skeletal muscle mass, WS = walking speed.

**Table 5:** Logistic regression analyses for osteoporosis with sarcopenia and its components among obesity subjects.

	Variables	Crude		Model 1		Model 2	
		OR (95% CI)	*P* value	OR (95% CI)	*P* value	OR (95% CI)	*P* value
Obesity Cases n* = 201* OP Cases n = 45	PBF (%)	1.00 (0.94–1.05)	.846	1.02 (0.94–1.10)	.696	0.98 (0.89–1.07)	.596
	ASMI (kg/m^2^)	0.97 (0.70–1.35)	.847	0.84 (0.50–1.42)	.511	0.95 (0.82–1.10)	.526
	Grip Strength (kg)	0.97 (0.93–1.01)	.145	0.99 (0.93–1.05)	.630	0.97 (0.91–1.04)	.355
	WS (m/s)	0.17 (0.05–0.61)	.007	1.30 (0.24–7.07)	.764	1.46 (0.24–8.87)	.679

Model 1 was adjusted for gender and age. Model 2 was adjusted for Model 1 variables, in addition to smoking and drinking habits, living conditions, physical activity, marital status and history of diseases.

BFM = body fat mass, PBF = percent body fat, SMI = skeletal muscle index, SMM = skeletal muscle mass, WS = walking speed.

## 4. Discussion

In this study, we found that the prevalence of osteoporosis was highest in the underweight group and gradually decreased with an increase in BMI, and higher PBF was significantly correlated with an increased risk of osteoporosis in normal weight and overweight subjects. Sarcopenia was associated with elevated odds of osteoporosis in normal-weight subjects independent of potential covariates. In normal-weight participants, a higher ASMI was associated with a reduced risk of osteoporosis.

### 4.1. Weight and osteoporosis

Several studies have shown a positive relationship between BMI and BMD, which is similar to our findings.^[4,27]^ Previous studies have reported that higher body weight appears to decelerate bone loss during menopause, and the beneficial effects of obesity on BMD can be attributed to the mechanical impact of body weight on bones.^[5,6]^ Animal-based investigations reveal that osteocytes possess a heightened sensitivity to biomechanical stress. Upon detection of shear stress signals by osteocytes, the release of sclerostin is inhibited, the activity of osteoclasts is diminished, and the differentiation of osteoblasts is stimulated. This substantiates the notion that an increase in body weight contributes to a favorable bone balance.^[4,28]^ In a comparable fashion, research encompassing 1988 young Chinese individuals and 4489 elderly Caucasians demonstrated a reverse association between body fat percentage and weight-adjusted bone mass, taking into account variables such as age, gender, height, menopausal status, and lifestyle factors,^[29]^ additionally, a study involving 1147 patients over 18 years of age revealed a negative correlation between body fat percentage and BMD, with adjustments for age, weight, height, ethnicity, and menopausal status.^[30]^ Therefore, not only passive loading but also muscle-induced strain is increased to decrease the risk of osteoporosis.^[31]^

In contrast, obesity, manifesting as a low-grade chronic inflammatory state, triggers the release of numerous cytokines detrimental to bone and muscle health, resulting in fatty infiltration of muscles and subsequently diminishing their strength and efficiency.^[32]^ Furthermore, obese individuals exhibit elevated levels of body fat and lean tissue mass, which have been linked to heightened circulating concentrations of proinflammatory cytokines. These cytokines have been associated with reduced BMD, accelerated bone deterioration, and an elevated risk of fractures among older adults.^[33]^ In our study, we found that higher PBF was significantly correlated with an increased risk of osteoporosis in normal-weight and overweight subjects, which is consistent with some previous studies.^[30,34]^

In this study, we found that the prevalence of osteoporosis was highest in the underweight group and gradually decreased with increasing BMI. The seeming obscurity surrounding the higher values in terms of BMD could be partially attributed to the well-established connection between estrogens and obesity. Studies reveal that obese postmenopausal women possess higher blood estrogen concentrations compared to their non-obese counterparts,^[29,35]^ which might elucidate the correlation between greater BMD and higher BMI among females. Nevertheless, estrogen levels alone do not solely govern bone mass, as numerous other factors also influence both bone and fat mass. High body fat is associated with inflammation and various adverse health outcomes, encompassing elevated mortality and metabolic disorders like diabetes and osteoporosis.^[20,36]^ Among the elderly, the relatively elevated visceral fat might have escalated the risk of osteoporosis, according to the body fat percentage. Individuals with high visceral fat exhibit a negative correlation with BMD due to augmented bone resorption triggered by proinflammatory cytokines and reduced bone formation stemming from insulin resistance and low insulin-like growth factor levels.^[37]^ Given the advantageous impact of bodily weight’s mechanical influence on bones, particularly from lean mass, the introduction of mechanical stimuli through exercise fosters pathways that aid in bone maintenance and growth.^[2]^

### 4.2. Sarcopenia and osteoporosis

Sarcopenia and osteoporosis, being 2 prevalent age-related disorders, frequently occur concurrently. Amidst the aging demographic, it is anticipated that the incidence of both these conditions will escalate in the forthcoming years, thereby heightening the vulnerability to fragility fractures, which are closely linked to considerable morbidity and mortality.^[38]^ In this study, we found that sarcopenia was associated with elevated odds of osteoporosis in normal-weight subjects independent of potential covariates. We did not find any coexistence of osteoporosis and sarcopenia in obese individuals as defined by BMI classification. Other methods of defining obesity may need to be introduced to more accurately assess the risk of osteoporosis and sarcopenia in different populations. Epidemiological investigations have revealed a positive correlation between osteoporosis, sarcopenia, and C-reactive protein (CRP), a biomarker indicating active inflammation.^[39]^ A systematic review and meta-analysis has underscored the significant prevalence of osteosarcopenia across various geriatric populations, regardless of the varying definitions of sarcopenia. Muscle tissue is widely acknowledged as the primary generator of anabolic mechanical stimuli for bone, and a reduction in mechanical loading contributes to diminished bone formation, ultimately resulting in fragile bone status.^[19]^ Numerous studies have indicated a connection between handgrip strength and a spectrum of health outcomes. Notably, a diminished level of handgrip strength is associated with a higher likelihood of multimorbidity in older adults, even after considering adjustments.^[40,41]^ Our study did not find a significant correlation between muscle function and osteoporosis, which may be due to the differences in muscle function assessment methods by sarcopenia. Our previous research has shown that when dynamic balance ability is used as a supplement to sarcopenia’s muscle function assessment, it has higher predictive value for osteoporosis.^[24]^ In a study conducted in vitro, researchers analyzed osteoblasts cultured with serum from participants categorized as normal, obese, obese/osteopenic, obese/sarcopenic, and obese/osteopenic/sarcopenic. Their findings revealed a diminished level of RunX2, a crucial transcription factor for osteoblast maturation, in all pathological groups when compared to healthy controls. However, notable changes in the osteoblast marker osteocalcin were observed only in obese participants, not in those with a combined obese, osteopenic, and sarcopenic status. This suggests that varying degrees of adipose tissue and muscle mass may influence bone biology.^[42]^

As this was a cross-sectional study, causality could not be evaluated. The participants of the current study were predominantly in a good health state, excluding those who were unable to engage in the annual national physical examination due to limitations (such as bedridden individuals or those with critical illnesses). Consequently, there is a likelihood that our findings underestimated the prevalence of osteoporosis and its related health consequences. Nevertheless, noteworthy disparities between participants with and without osteoporosis remained evident, indicating that statistical power was unlikely to be a significant concern. Further longitudinal follow-up studies are required.

## 5. Conclusions

In this study, we found that the prevalence of osteoporosis was highest in the underweight group and gradually decreased with increasing BMI. Sarcopenia, PBF, and ASMI were associated with elevated odds of osteoporosis in normal-weight subjects independent of potential covariates, and a higher PBF was associated with an increased risk of osteoporosis in overweight people, no such association was found in other weight groups. Different amounts of adipose tissue and muscle mass may alter bone biology. Further longitudinal follow-up studies are required.

## Acknowledgments

We would like to thank all participants and co-researchers who participated in this study.

## Author contributions

**Data curation:** Xing Yu, Yaqing Zheng.

**Formal analysis:** Xing Yu.

**Investigation:** Xing Yu, Yaqing Zheng, Yuewen Liu, Peipei Han, Xiaoyu Chen, Naiwen Zhang, Yejia Ni, Ziyi Zhou.

**Methodology:** Xing Yu, Yaqing Zheng, Qi Guo.

**Writing – original draft:** Xing Yu, Yaqing Zheng.

**Writing – review & editing:** Xing Yu, Yaqing Zheng, Qi Guo.

## Supplementary Material

**Figure:**
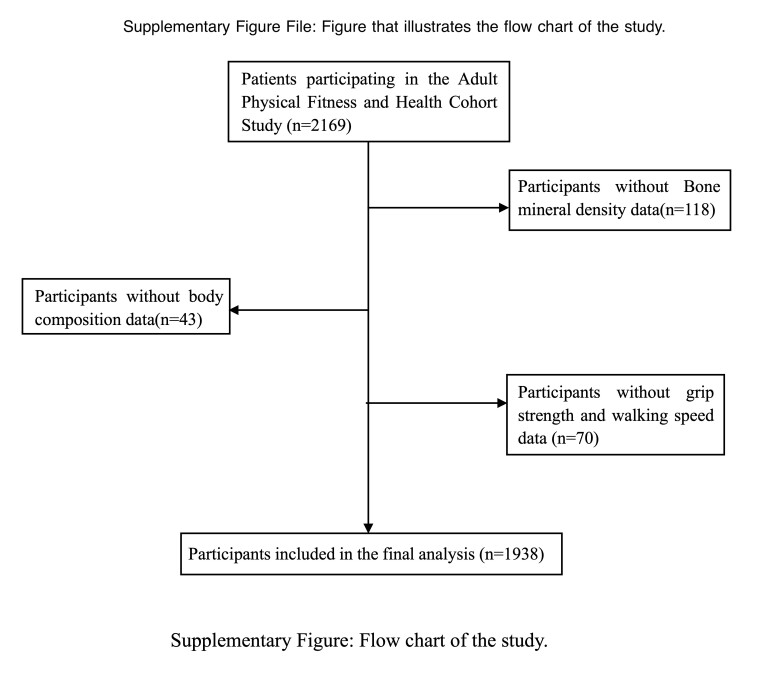


## References

[1] Cauley JA Giangregorio L. Physical activity and skeletal health in adults. Lancet Diabetes Endocrinol. 2020;8:150–62. 31759956 10.1016/S2213-8587(19)30351-1

[2] Pagnotti GM Styner M Uzer G. Combating osteoporosis and obesity with exercise: leveraging cell mechanosensitivity. Nat Rev Endocrinol. 2019;15:339–55. 30814687 10.1038/s41574-019-0170-1 PMC6520125

[3] Rosenberg IH. Sarcopenia: origins and clinical relevance. Clin Geriatr Med. 2011;27:337–9. 21824550 10.1016/j.cger.2011.03.003

[4] Pinar-Gutierrez A Garcia-Fontana C Garcia-Fontana B. Obesity and bone health: a complex relationship. Int J Mol Sci. 2022;23:8303. 35955431 10.3390/ijms23158303 PMC9368241

[5] Evans AL Paggiosi MA Eastell R Walsh JS. Bone density, microstructure and strength in obese and normal weight men and women in younger and older adulthood. J Bone Miner Res. 2015;30:920–8. 25400253 10.1002/jbmr.2407

[6] Fang J Gao J Gong H Zhang T Zhang R Zhan B. Multiscale experimental study on the effects of different weight-bearing levels during moderate treadmill exercise on bone quality in growing female rats. Biomed Eng Online. 2019;18:33. 30902108 10.1186/s12938-019-0654-1 PMC6431042

[7] Garnero P Sornay-Rendu E Claustrat B Delmas PD. Biochemical markers of bone turnover, endogenous hormones and the risk of fractures in postmenopausal women: the OFELY study. J Bone Miner Res. 2000;15:1526–36. 10934651 10.1359/jbmr.2000.15.8.1526

[8] Gkastaris K Goulis DG Potoupnis M Anastasilakis AD Kapetanos G. Obesity, osteoporosis and bone metabolism. J Musculoskelet Neuronal Interact. 2020;20:372–81. 32877973 PMC7493444

[9] Ilesanmi-Oyelere BL Coad J Roy N Kruger MC. Lean body mass in the prediction of bone mineral density in postmenopausal women. Biores Open Access. 2018;7:150–8. 30327744 10.1089/biores.2018.0025 PMC6188582

[10] Kang D Liu Z Wang Y. Relationship of body composition with bone mineral density in northern Chinese men by body mass index levels. J Endocrinol Invest. 2014;37:359–67. 24477947 10.1007/s40618-013-0037-6

[11] Rexhepi S Bahtiri E Rexhepi M Sahatciu-Meka V Rexhepi B. Association of body weight and Body Mass Index with bone mineral density in women and men from Kosovo. Mater Sociomed. 2015;27:259–62. 26543419 10.5455/msm.2015.27.259-262 PMC4610606

[12] Riggs BL. The mechanisms of estrogen regulation of bone resorption. J Clin Invest. 2000;106:1203–4. 11086020 10.1172/JCI11468 PMC381441

[13] Hart DJ Spector TD. The relationship of obesity, fat distribution and osteoarthritis in women in the general population: the Chingford Study. J Rheumatol. 1993;20:331–5. 8474072

[14] Adler BJ Kaushansky K Rubin CT. Obesity-driven disruption of haematopoiesis and the bone marrow niche. Nat Rev Endocrinol. 2014;10:737–48. 25311396 10.1038/nrendo.2014.169

[15] Kennedy DE Witte PL Knight KL. Bone marrow fat and the decline of B lymphopoiesis in rabbits. Dev Comp Immunol. 2016;58:30–9. 26577994 10.1016/j.dci.2015.11.003 PMC4775299

[16] Vincent HK Raiser SN Vincent KR. The aging musculoskeletal system and obesity-related considerations with exercise. Ageing Res Rev. 2012;11:361–73. 22440321 10.1016/j.arr.2012.03.002 PMC3356456

[17] Lee DY Shin S. Association of sarcopenia with osteopenia and osteoporosis in community-dwelling older Korean Adults: a cross-sectional study. J Clin Med. 2021;11:129. 35011870 10.3390/jcm11010129 PMC8745168

[18] Liu C Liu N Xia Y Zhao Z Xiao T Li H. Osteoporosis and sarcopenia-related traits: a bi-directional Mendelian randomization study. Front Endocrinol (Lausanne). 2022;13:975647. 36187130 10.3389/fendo.2022.975647 PMC9515352

[19] Nielsen BR Abdulla J Andersen HE Schwarz P Suetta C. Sarcopenia and osteoporosis in older people: a systematic review and meta-analysis. Eur Geriatr Med. 2018;9:419–34. 34674498 10.1007/s41999-018-0079-6

[20] Lee S Ko K Shin S Park HS Hong N Rhee Y. Adipopenia is associated with osteoporosis in community-dwelling non-underweight adults independent of sarcopenia. Arch Osteoporos. 2023;18:44. 36949274 10.1007/s11657-023-01233-x

[21] Tanaka S Kuroda T Saito M Shiraki M. Overweight/obesity and underweight are both risk factors for osteoporotic fractures at different sites in Japanese postmenopausal women. Osteoporos Int. 2013;24:69–76. 23229467 10.1007/s00198-012-2209-1

[22] Cruz-Jentoft AJ Baeyens JP Bauer JM.; European Working Group on Sarcopenia in Older People. Sarcopenia: European consensus on definition and diagnosis: report of the European Working Group on Sarcopenia in Older People. Age Ageing. 2010;39:412–23. 20392703 10.1093/ageing/afq034 PMC2886201

[23] Zamboni M Rubele S Rossi AP. Sarcopenia and obesity. Curr Opin Clin Nutr Metab Care. 2019;22:13–9. 30461451 10.1097/MCO.0000000000000519

[24] YuXHouLGuoJ. Combined effect of osteoporosis and poor dynamic balance on the incidence of sarcopenia in elderly Chinese Community Suburban-Dwelling Individuals. J Nutr Health Aging. 2020;24:71–7.31886811 10.1007/s12603-019-1295-6

[25] Wang J Zhang Y Yang Y. The prevalence and independent influencing factors of obesity and underweight in patients with schizophrenia: a multicentre cross-sectional study. Eat Weight Disord. 2021;26:1365–74. 32557379 10.1007/s40519-020-00920-9

[26] Craig CL Marshall AL Sjostrom M. International physical activity questionnaire: 12-country reliability and validity. Med Sci Sports Exerc. 2003;35:1381–95. 12900694 10.1249/01.MSS.0000078924.61453.FB

[27] Sukumar D Schlussel Y Riedt CS Gordon C Stahl T Shapses SA. Obesity alters cortical and trabecular bone density and geometry in women. Osteoporos Int. 2011;22:635–45. 20533027 10.1007/s00198-010-1305-3 PMC2994953

[28] Tan SD De Vries TJ Kuijpers-Jagtman AM Semeins CM Everts V Klein-Nulend J. Osteocytes subjected to fluid flow inhibit osteoclast formation and bone resorption. Bone. 2007;41:745–51. 17855178 10.1016/j.bone.2007.07.019

[29] Zhao LJ Jiang H Papasian CJ. Correlation of obesity and osteoporosis: effect of fat mass on the determination of osteoporosis. J Bone Miner Res. 2008;23:17–29. 17784844 10.1359/JBMR.070813 PMC2663586

[30] Lu H Fu X Ma X. Relationships of percent body fat and percent trunk fat with bone mineral density among Chinese, black, and white subjects. Osteoporos Int. 2011;22:3029–35. 21243336 10.1007/s00198-010-1522-9

[31] Fassio A Idolazzi L Rossini M. The obesity paradox and osteoporosis. Eat Weight Disord. 2018;23:293–302. 29637521 10.1007/s40519-018-0505-2

[32] Goisser S Kemmler W Porzel S. Sarcopenic obesity and complex interventions with nutrition and exercise in community-dwelling older persons--a narrative review. Clin Interv Aging. 2015;10:1267–82. 26346071 10.2147/CIA.S82454 PMC4531044

[33] Cauley JA Danielson ME Boudreau RM.; Health ABC Study. Inflammatory markers and incident fracture risk in older men and women: the health aging and body composition study. J Bone Miner Res. 2007;22:1088–95. 17419681 10.1359/jbmr.070409

[34] Zhao LJ Liu YJ Liu PY Hamilton J Recker RR Deng H-W. Relationship of obesity with osteoporosis. J Clin Endocrinol Metab. 2007;92:1640–6. 17299077 10.1210/jc.2006-0572 PMC1868430

[35] Reid IR. Relationships between fat and bone. Osteoporos Int. 2008;19:595–606. 17965817 10.1007/s00198-007-0492-z

[36] Rhee EJ. The influence of obesity and metabolic health on vascular health. Endocrinol Metab (Seoul). 2022;37:1–8. 35255597 10.3803/EnM.2022.101 PMC8901957

[37] Zhu K Hunter M James A Lim EM Cooke BR Walsh JP. Relationship between visceral adipose tissue and bone mineral density in Australian baby boomers. Osteoporos Int. 2020;31:2439–48. 32719992 10.1007/s00198-020-05556-0

[38] Edwards MH Dennison EM Aihie Sayer A Fielding R Cooper C. Osteoporosis and sarcopenia in older age. Bone. 2015;80:126–30. 25886902 10.1016/j.bone.2015.04.016 PMC4601530

[39] Clynes MA Gregson CL Bruyere O. Osteosarcopenia: where osteoporosis and sarcopenia collide. Rheumatology (Oxford). 2021;60:529–37. 33276373 10.1093/rheumatology/keaa755

[40] Zhao X Chen S Liu N Hu F Yu J. Handgrip strength is positively associated with successful aging in older adults: a national cross-sectional study in China. J Affect Disord. 2023;333:30–7. 37084959 10.1016/j.jad.2023.04.041

[41] Zhao X Zhang H Yu J Wang J. Association of possible sarcopenia with major chronic diseases and multimorbidity among middle-aged and older adults: Findings from a national cross-sectional study in China. Geriatr Gerontol Int. 2023;23:925–31. 37915295 10.1111/ggi.14720

[42] Wannenes F Papa V Greco EA. Abdominal fat and sarcopenia in women significantly alter osteoblasts homeostasis in vitro by a WNT/ beta -Catenin Dependent Mechanism. Int J Endocrinol. 2014;2014:278316. 24963291 10.1155/2014/278316 PMC4054618

